# The impact of organ transplantation on changes in the oral microbiome, particularly on bacteria causing periodontal inflammation - narrative review

**DOI:** 10.3389/fcimb.2026.1859519

**Published:** 2026-07-29

**Authors:** Iwona Ordyniec-Kwaśnica, Damian Muszyński, Bartosz Słomiński, Mateusz Lampkowski, Anna Kudra

**Affiliations:** 1 Division of Dental Prosthetics, Medical University of Gdańsk, Gdansk, Poland; 2 Pomeranian Dental Center Kryspin-Dent, Gdansk, Poland; 3 Department of Medical Immunology, Medical University of Gdańsk, Gdansk, Poland; 4 ”Zdrowie” LLC DENTUS Dental Clinics in Gdynia, Gdynia, Poland

**Keywords:** dysbiosis, oral health, oral microbiome, periodontal inflammation, transplantation

## Abstract

With the development of technology and medicine, more and more diseases in today’s world are becoming curable. Ultimately, when there is no improvement in health and a deterioration in the efficiency or functionality of a given organ, organ transplantation becomes the only option. In 2024 alone, data from the Global Observatory on Donation and Transplantation indicate that over 173, 000 organ transplants were performed worldwide, reflecting a consistent upward trend in recent years The introduction of a “foreign” organ into the body is not without consequences. Clinical manifestations may vary, including, among others, transplant rejection. These alterations may also extend to highly sensitive biological environments such as the oral microbiome, particularly affecting bacterial populations implicated in gum/gingival inflammation. This publication shows what microbiological changes can be caused by the transplantation of certain organs and whether, in general, every transplant means the same change in the oral microbiota.

## 1 Introduction

The history of modern transplantology dates back to the turn of the 19th and 20th centuries, which at that time was met with repeated opposition (Schlich, 2024). One of the first organs to be successfully transplanted was the kidney. Although it ceased to function after a few days, this was the first major step toward the development of this field of medicine (Hatzinger et al., 2016). At that time, despite significant medical progress, there were still cases of what is commonly referred to as “transplant rejection.” The recipient’s T lymphocytes may recognize the transplanted tissue as non-self, thereby initiating a cascade of immunological effector mechanisms (Shlomchik, 2007). All kinds of post-transplant disorders can affect the oral microbiome (Campbell et al., 2023).

Currently, it is believed that the oral microbiome plays a major role in maintaining homeostasis in the oral cavity and, in case of dysbiosis, may lead to the development of periodontal disease (Aas et al., 2005; Socransky and Haffajee, 2005; Dewhirst et al., 2010). Periodontitis, a notorious chronic disease affecting periodontal tissues, ranks among the most prevalent chronic conditions. Two of the teeth’s supporting structures, the periodontal ligament and alveolar bone, are gradually destroyed in this complex, chronic inflammatory disease, which results in the formation of periodontal pockets, loss of connective tissue attachment, and the resorption of alveolar bone (de Molon et al., 2018; Kure et al., 2019). When left untreated, periodontitis may have local as well as systemic consequences, such as tooth loss affecting patients’ ability to grind food, speak clearly, but also lowers their self-esteem and may contribute to progression to other bacteria-related diseases (Nagpal et al., 2015; Hong et al., 2025). The aim of this narrative review was to summarize the current knowledge regarding alterations in the oral microbiome in patients who have undergone kidney, liver, heart, or lung transplantation, with particular emphasis on microorganisms associated with periodontal disease and their potential implications for oral health.

### 1.1 Methods

A narrative literature review was conducted to investigate the association between organ transplantation and modifications in the oral microbiome, with a particular focus on microorganisms associated with periodontal disease. The following keywords and combinations of terms were used: “oral microbiome”, “oral microbiota”, “periodontal disease”, “periodontitis”, “organ transplantation”, “kidney transplantation”, “liver transplantation”, “heart transplantation”, “lung transplantation”, and “oral health”. The literature search was conducted using the PubMed and Google Scholar databases and included studies published up to February 2026. Studies published in English that investigated oral microbiome changes following kidney, liver, heart, and lung transplantation were evaluated. Original research articles, clinical studies, experimental studies, systematic reviews, and narrative reviews were included. Publications focusing exclusively on transplantation outcomes unrelated to oral health or microbiological alterations were excluded. Particular attention was given to studies evaluating changes in bacterial composition, microbial diversity, dysbiosis, and the potential impact of transplant-associated microbiological alterations on periodontal health.

## 2 Oral microbiome in homeostasis

The human oral cavity is an optimal habitat for a huge variety of microorganisms, including not only bacteria and fungi but also protozoa, viruses, and archaea (Zorba et al., 2020). Environment in the oral cavity, consisting of different habitats like tongue, teeth, gingival sulcus, cheeks, soft and hard palates and tonsils, creates optimal conditions for growth and colonization via its stable temperature, high humidity and constant delivery of nutrients. According to the expanded Human Oral Microbiome Database (eHOMD) oral cavity is colonized with 774 oral bacterial species (Verma et al., 2018; Zorba et al., 2020). Using 16S rDNA profiling, six extensive phyla have been distinguished: *Firmicutes, Actinobacteria, Proteobacteria, Fusobacteria, Bacteroidetes*, and *Spirochaetes* (Verma et al., 2018). Although these bacteria are a part of the oral cavity’s microbiome, more recent research has concentrated on their association with several illnesses, including periodontitis, gingivitis, and dental caries, all of which are clearly linked to changes in the oral microbiota (Willis and Gabaldón, 2020).

Microorganisms within the oral microbiome function as an interactive community, coordinating their activity through interspecies interactions rather than behaving as independent cells. Horizontal gene transfer, interference in signaling processes to gain an edge over others during colonization, competitiveness with other microbial organisms, and synergistic metabolic interactions or competition for resources and habitats are the main oral microbial interactions (Marsh, 2005; Marsh and Zaura, 2017).

The study conducted in 2018 by Lima. et al. demonstrated that *S. aureus* may engage in cooperation with, pathogen typical for the oral microbiome, *F. nucleatum*, to directly bind to the oral *oral microbiome* and alter it. Differential expression of the S. aureus global regulator sarA, which is linked to the reduction of pathogenicity, biofilm formation, and susceptibility to antibiotic treatment, is caused by specific contact with the oral bacterium *F. nucleatum* (Lima et al., 2018). Another study showed interesting data about the “community selection” effect occurring in mice’s oral microbial community. Using *Escherichia coli* as a model intestinal bacterium and *in vitro* microbial floras that represent microorganisms that live in mice’s oral cavities and digestive tracts, researchers showed that *E. coli* exhibits a remarkable community preference. It didn’t perform well in the microbial flora of mice’s oral cavity, but it excelled when placed in the intestinal microbial community. Researchers concluded that the oral community detects the LPS of *E. coli* and uses oxygen-free radicals to eliminate the bacteria (He et al., 2010).

## 3 Oral microbiome in periodontitis

### 3.1 Dental biofilm

Periodontitis, an inflammatory chronic disease of periodontal tissues, is closely connected to dysbiosis in the bacterial ecosystem of the oral cavity (Haffajee and Socransky, 1994; Li et al., 2023). When a synergetic relationship between the host’s oral cavity and bacteria becomes compromised, it may lead to the build-up of dental biofilm. It mostly happens due to variation of different factors such as sugar-enriched diet, poor hygiene, compromised function of salivary glands causing xerostomia and impaired immune function (Haffajee and Socransky, 1994; Chenicheri et al., 2017; Sotozono et al., 2021). The base unit of biofilm, a complex multi-microbial community, is a cluster or microcolony which is characterized as a discrete group of bacterial cells, from one species or several, enclosed in the matrix with salivary polymers as well as bacterial extracellular products. Adhesion mediated by several glycoproteins allows bacteria to evade the host’s defenses and promotes colonization of mucosal surfaces (Chenicheri et al., 2017), the development of dental biofilms takes three stages. Firstly, a specific surface in the oral cavity gets colonized by planktonic forms which attach to it irreversibly. Steadily multiplying, they produce their own exopolymeric matrix which allows them to create a constantly growing, sessile, mixed population (Lawrence et al., 1991). Bacteria that would not be able to adhere to a smooth, even surface can now do so once a base has been formed and adhered to the colonized surface. At the ultrastructural level, biofilms exhibit a highly organized three-dimensional architecture composed of microcolonies embedded within an exopolymeric matrix. These microcolonies are separated by interstitial channels that enable the diffusion of oxygen, nutrients, and metabolic by-products. In this configuration, bacteria favoring an anaerobic environment will occupy centric regions of biofilms, where condensation is high, and oxygen levels are low, whereas external areas will be dominated by bacteria living in an aerobic environment (Lawrence et al., 1991; Nadell et al., 2009).

### 3.2 Impact on periodontal tissue

In periodontitis a shift occurs in sub-gingival biofilm from a healthy diverse commensal community to state of dysbiosis where opportunistic “red complex” bacteria (*Porphyromonas gingivalis, Tannerella forsythia, Treponema denticola*) overtake this niche habitat leading to imbalance in the microbial composition of biofilms, which stimulates the host’s immunological response, resulting in a chronic inflammatory state (Könönen et al., 2019). The extracellular polymeric matrix surrounding the polymicrobial community confers resistance to antimicrobial agents and limits accessibility to host immune defenses (Schenkein et al., 2020). Bacteria in these biofilms may have direct or indirect pathogenic mechanisms causing distress to gingival tissue. Direct mechanism is more focused on bacterial virulence factors like secreted bioactive compounds such as exoenzymes, toxins, and metabolic end products that cause harm to cells and intercellular matrix of the host connective tissue (Zhang et al., 2024). Furthermore, these bacteria biological components such as lipopolysaccharide (Gram-negative bacteria), peptidoglycan, lipoteichoic acid (Gram-positive bacteria), nucleic acids, flagella, fimbriae, outer membrane proteins (OMPs), vesicles (OMVs) and exopolysaccharides may trigger hosts immune response when present in surrounding tissue. Upon exposure to bacterial components, both immune and non-immune host cells are induced to produce a spectrum of inflammatory mediators, including cytokines and prostaglandins. Released mediators contribute to causing one of the characteristic symptoms of periodontitis, by enhancing the process of bone tissue resorption (Cekici et al., 2014; Di Stefano et al., 2022).

**Figure 1:**
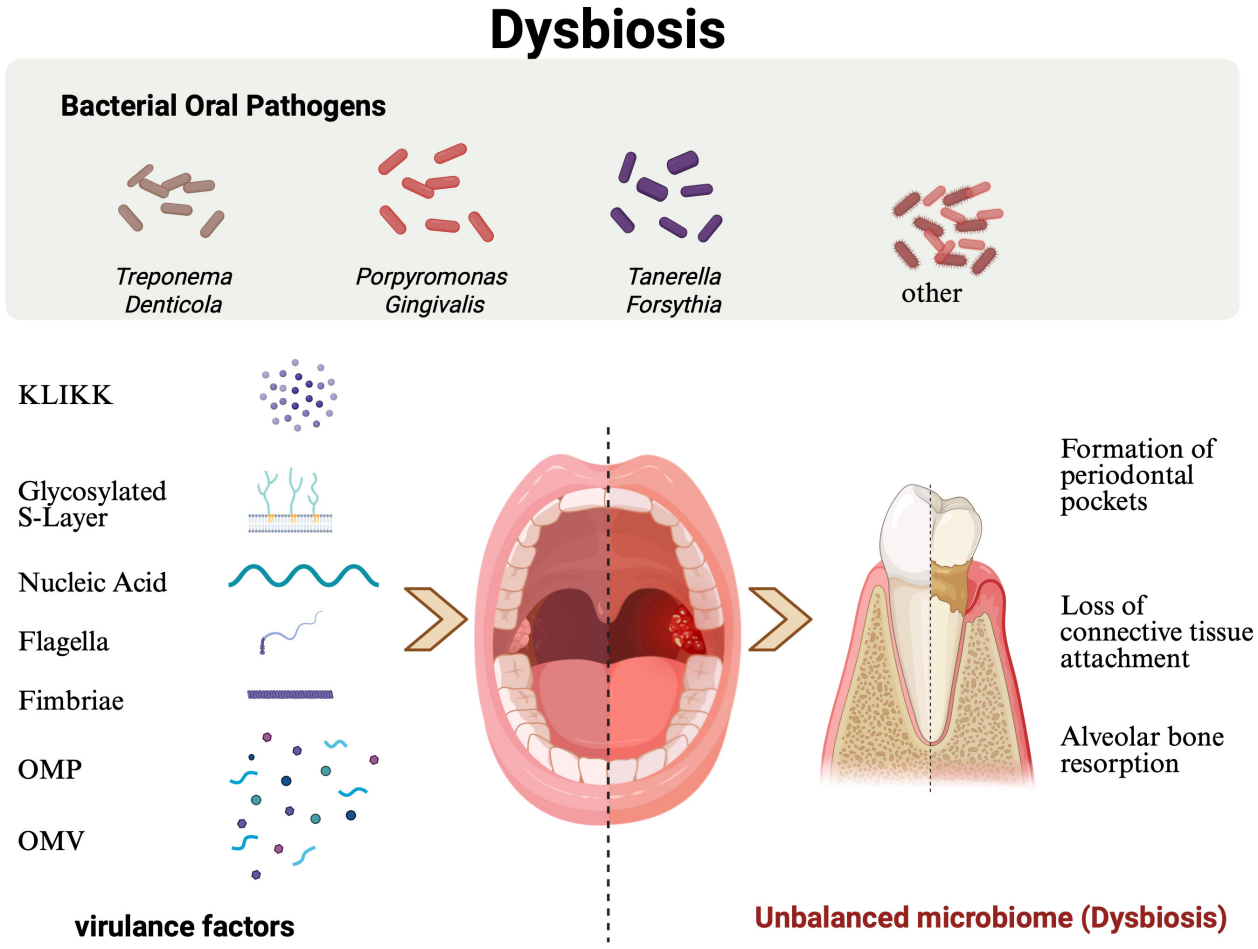
Virulance factors influencing process of dysbiosis. The illustration was created on BioRender.com.

A summary of the above information is presented in **Figure 1**.

## 4 Oral pathogens and their virulence factors in periodontitis development

Dysbiosis refers to a pathogenic condition in which bacteria inflict illness on the host. A persistent cycle of dysbiosis, immunological response, and tissue degradation is caused by inflammation, which is a major factor in changing the microbial population. In inflamed periodontal pockets, conditions such as reduced oxygen tension, increased availability of host-derived protein substrates, and elevated gingival crevicular fluid flow favor the proliferation of inflammophilic, anaerobic, proteolytic microorganisms, further supported by synergistic microbial interactions (Kawamoto et al., 2021). Overgrowth of pathogenic bacteria belonging the red complex is the most apparent indication of dysbiosis. Although the subgingival microbiome within the healthy periodontal state comprises those conventionally described oral pathogens, they do not activate any immune responses due to their relatively low population in the good health state. In periodontitis, bacteria that are not prevalent in a healthy condition increase significantly in number and synergize, leading to development of pathological state (Qin et al., 2022). Bacterial virulence factors are central to the initiation and progression of periodontitis, acting synergistically to disrupt host homeostasis and promote tissue destruction.

One of the most recognizable bacteria included in the red complex is *T. forsythia.* This specific Gram-negative bacterium is an anaerobe belonging to the *Porphyromonadaceae* family (Posch et al., 2012). *T. forsythia* growth and development, because of its asachrolytic physiology, is dependent on availability of amino acids in its surroundings. Therefore, *T. forsythia* secretes at least two different proteolytic enzymes, presumably to participate in the degradation of host proteins (Grenier, 1995; Saito et al., 1997). According to observations made by Ksiazek et al., these proteins, known as KLIKK proteases, may be key virulence factors. Partially purified recombinant proteases were introduced to protein substrates, including collagen, gelatin, elastin, and casein in zymografic gels resulting in different levels of proteolytic activity. This led researchers to conclusion that active proteases secreted by *T. forsythia* may lead to degradation of hosts protein to easily accessible amino acids (Ksiazek et al., 2015). Another virulence factor presented by *T. forsythia* is its glycosylated S-layer mentioned in Sakakibara et al. Study. It was stated that said S-layer may be essential in early stages of periodontal diseases, since it assist in the attachment and invasion of human epithelial cancer cells, such as KB cells and gingival cancer cells (Ca9–22) (Sakakibara et al., 2007).

## 5 Transplants

As mentioned earlier, transplantation may induce alterations in the oral microbiome, which can contribute to the development of periodontal disease (Gao et al., 2024). Below are descriptions of the most common transplants and microbiotic changes, and thus the risk of periodontal disease.

### 5.1 Kidney transplantation

Kidney transplantation is one of the most frequently performed solid-organ transplant procedures and is associated with profound immunological and metabolic changes that may influence the oral microbiome. As a result of a kidney transplant, changes in the oral microbiome may theoretically occur in the recipient. This information was included, for example, by Xiang et al. The study population comprised five healthy control subjects and eleven kidney transplant recipients. In the transplant group, saliva samples were collected longitudinally at five postoperative time points and subjected to subsequent analysis. A significant increase in the number of bacteria in groups such as *Actinobacteria, Tenericutes*, and *Spirochaetes* was found in transplant recipients. However, after 14 days, the number of species began to gradually decrease, and the oral microbiome became more stable (Xiang et al., 2023). From a periodontal perspective, it seems important to note that the bacteria mentioned above, or more precisely their representatives, can be isolated in saliva samples from people suffering from periodontal inflammation (Lundmark et al., 2019).

Qu et al. analyzed the correlation between gut and oral microbiome profiles and their dynamics following kidney transplantation. Twenty-six patients who were scheduled to undergo kidney transplantation were enrolled in the study. Saliva samples were collected from them, as in the previous study, as well as stool samples. The material was analyzed. In saliva, the dominant bacteria were *Firmicutes, Proteobacteria, Actinobacteria*, and *Bacterioidetes*. However, here too, it was observed that after a period of time, there was a decrease in the species diversity of the oral cavity. However, this study demonstrated a shift in the composition of the oral microbiota (Qu et al., 2025).

Changes in the microbiome after kidney transplantation were also studied by Xiang et al. In this case, the study was conducted in the context of delayed transplant function. The participants were 40 patients who had undergone kidney transplantation, from whom saliva was also collected and analyzed. The authors concluded that salivary microbiota composition exhibited temporal changes after transplantation, in both immediate and delayed graft function cohorts. Particular attention should be paid to changes in the amounts of *Bacterioidetes* and *Veillonella* bacteria (belonging to *Firmicutes*) (Xiang et al., 2024*)*.

Kidney transplantation causes changes in the oral microbiome, which are characterized by quantitative and qualitative changes. However, over time, it stabilizes. These studies show that the main bacteria affected by these changes belong to *Bacterioidetes, Firmicutes*, and *Actinobacteria*. *Actinobacteria* have already been mentioned as bacteria that can cause periodontitis (Lundmark et al., 2019). However, according to other sources, their number is higher in patients with healthy oral tissues than in those with periodontitis. *Bacterioidetes* and *Firmicutes* may also contribute to periodontitis (Abusleme et al., 2013). It is also worth noting that *Firmicutes* may also be responsible for caries in the oral cavity (Fakhruddin et al., 2019).

### 5.2 Liver transplantation

The liver is a multifunctional organ involved in metabolism and immune regulation. Liver transplantation remains the treatment of choice for end-stage liver disease and may be associated with alterations in microbial communities. Babu et al. conducted one of the key studies in this area, involving 94 liver transplant recipients with cirrhosis and a donor group. Salivary samples were obtained pre-transplantation from both groups and post-transplantation from recipients, followed by microbiological analysis. During the observation, an increase in *Bacillota* (*Firmicutes*), *Bacterioidetes*, and *Actinomycetota* bacteria was noted. An increased amount of these bacteria was observed in particular among patients who subsequently developed rejection, biliary complications, or infection. This study shows that the bacteria responsible for the development of periodontal inflammation may also contribute to disorders that can ultimately lead to more or less serious complications (Babu et al., 2026).

The relationship between liver transplantation and oral alterations was investigated by Norrman et al. The study included 84 adult liver transplant recipients. Participants were stratified according to the presence of diabetes, periodontal status (including disease severity), and immunosuppressive therapy. Although microbiological analysis was not a primary objective of the study, no significant quantitative or qualitative differences were observed between patients with and without diabetes. Notably, diabetes was not associated with differences in levels of biomarkers indicative of periodontal inflammation, despite being linked to poorer periodontal status (Norrman et al., 2021).

The studies presented above show that changes in the microbiome after liver transplantation may or may not occur. If these changes do occur, they are similar to those that occur in kidney transplantation, i.e., an increased risk of periodontitis but also potential tooth decay (Xiang et al., 2023; Qu et al., 2025). There is little research related to the oral microbiome in this case, but there is much more on the correlation between the gut microbiome and liver transplantation. The authors point out, in particular, the enrichment of the gut microbiome with bacteria of the genera *Clostridiales, Streptococcus*, and *Enterococcus* (Wirth et al., 2023). It is important to note that *Streptococcus* are representatives of *Firmicutes*, which were already mentioned in the first study (Megrian et al., 2020). This would mean that liver transplantation may indeed have an impact on microbiological changes in this direction. Further research is needed to clearly determine the correlation between the oral microbiome and liver transplantation.

### 5.3 Heart transplantation

Heart transplantation is a life-saving treatment for end-stage heart failure and may influence oral microbial composition through immunosuppressive therapy and systemic physiological changes. Heart transplantation may influence changes in the oral microbiome. These conclusions were reported by Olek et al. The study included 25 patients who had a history of heart transplantation. The study analyzed the oral health of the patients, paying particular attention to pathogens that could potentially cause *periimplantitis*. The specific microorganisms that were identified were *Aggregatibacter actinomycetemcomitans, Porphyromonas gingivalis, Treponema denticola*, and *Candida albicans*. It was also noted that the overall oral health of these patients was not satisfactory (Olek et al., 2022).

An additional study on this topic was conducted by Jones et al. The study included 30 pediatric heart transplant recipients and 30 healthy controls. A dental examination was performed in both groups. The results demonstrated no significant differences in overall oral health status between the groups. However, the transplant group exhibited a higher prevalence of pathogenic oral microbiota, poorer oral hygiene, and a higher DMFT (Decayed, Missing, Filled Teeth) index (Kuramitsu and Wang, 2006; Yamashita and Takeshita, 2017).

Changes resulting from heart transplantation may affect not only the oral microbiome, but also the gut microbiome. Heart transplantation and therapies using devices that support the left ventricle are procedures performed in patients with severe heart failure. However, despite technological advances, complications such as inflammation and even progression of heart failure still occur (Yuzefpolskaya et al., 2023). This suggests that since the changes affect the oral cavity with simultaneous inflammation, it is possible that bacteria responsible for periodontal inflammation are involved in this condition (Plachokova et al., 2021).

In the case of heart transplantation, in addition to bacteria responsible for the potential development of periodontitis (*Aggregatibacter actinomycetemcomitans, Porphyromonas gingivalis, Treponema denticola*), *Candida albicans* also appears (Farias et al., 2012; Olek et al., 2022). As a typical fungal pathogen, it commonly manifests in the oral cavity of immunocompromised individuals, ultimately resulting in oral candidiasis (Sampaio-Maia et al., 2022). These findings suggest that, alongside periodontitis—an important comorbidity in transplant recipients—the range of relevant oral pathological conditions should also encompass an increased risk of dental caries and oral candidiasis.

### 5.4 Lung transplantation

Because of the close anatomical connection between the respiratory tract and oral cavity, lung transplantation may have unique effects on oral and respiratory microbial communities. Unfortunately, there is little research specifically on the oral microbiome and lung transplantation. One of the few examples of a study focusing on the upper respiratory tract as a whole is the study conducted by Cai et al. A series of samples were collected from 51 people after lung transplantation, including bronchoalveolar lavage fluid, sputum, and, most importantly, throat swabs. *Streptococcus, Prevotella*, and *Staphylococcus* were the most dominant species in the throat microbiome. Furthermore, this microbiome was the most diverse in terms of both internal and external aspects and was considered one of the most dynamic (Cai et al., 2025).

Unlike the previously discussed transplant contexts, lung transplantation involves an organ directly connected to the upper respiratory tract and, indirectly, the oral cavity. This continuity facilitates microaspiration of oral microbiota into the lower airways, potentially influencing the composition of the pulmonary microbiome. Studies report that patients who have undergone such a procedure have an enriched flora in the lungs with representatives of *Prevotella* bacteria, a typical representative of the oropharyngeal area (McGinniss et al., 2022; Ponholzer et al., 2024). Furthermore, changes in the *Prevotella/Streptococcus* ratio have been observed in patients who experienced primary graft dysfunction (McGinniss et al., 2022). It may be hypothesized that the oropharyngeal microbiome has an adverse effect on transplant acceptance by the recipient. This is confirmed by subsequent studies. Natalini et al. investigated the association between the lower respiratory tract microbiome and lung transplantation outcomes. To this end, 103 lung transplant recipients were included in the study, and those who developed acute cellular rejection were selected. Samples of the local microbiome were collected and analyzed. The results showed a lower enrichment of the lower respiratory tract with representatives of the oral microbiome, such as *Prevotella* and *Veillonella* (Natalini et al., 2024).

Changes in the oral cavity caused by lung transplantation cannot be clearly determined. The information gathered suggests that there may be a potential increase in the number of Streptococcus and *Prevotella* species, the growth of which correlates with an increased risk of periodontal inflammation, but also an increased risk of tooth decay (Karched et al., 2022; Cai et al., 2025; Zhou et al., 2025). The oral microbiome can also be transferred, most likely through the respiratory tract, to the transplanted lungs, thereby increasing the risk of post-transplant disorders. An interesting fact is that the bacteria most commonly found are also the bacteria responsible for periodontal inflammation in the oral cavity (Natalini et al., 2024; Rohmah et al., 2025). Further research is needed in this area to determine the specific impact of lung transplantation on the oral microbiome.

A summary of the above information is presented in **Table 1**.

**Table 1:** Comparison of the impact of individual organ transplants in terms of their effect on the oral microbiome.

Transplantable organ	Sources	Changes in the microbiome responsible for periodontal inflammation	Other observed changes in the oral microbiome
Kidney	(Xiang et al., 2024; Qu et al., 2025)	increase in the number of *Bacterioidetes, Firmicutes, Actinobacteria*	increase in the number of *Proteobacteria*
Liver	(Babu et al., 2026)	increase in the number of *Bacillota* (*Firmicutes*), *Bacterioidetes*, and *Actinomycetota*	_
Heart	(Olek et al., 2022)	increase in the number of *Aggregatibacter actinomycetencomitans, Porphyromonas gingivalis, Treponema denticola*	increase in the number of *Candida albicans*
Lung	(Natalini et al., 2024; Cai et al., 2025)	theoretical increase in *Streptococcus, Prevotella*	“migration” of the oral microbiome to transplanted lungs, particularly *Veillonella*

### 5.5 Factors influencing oral microbiome alterations after transplantation

The interpretation of microbiological changes observed following organ transplantation should be approached with caution, as several confounding factors may influence the oral microbiome independently of the transplantation procedure itself. Immunosuppressive therapy, routinely administered to prevent graft rejection, may alter host immune responses and facilitate colonization by opportunistic microorganisms, thereby contributing to microbial dysbiosis. In addition, perioperative and long-term antibiotic therapy can substantially modify microbial community composition, reduce microbial diversity, and affect the resilience of the oral microbiome (Zaura et al., 2015). Systemic diseases frequently observed in transplant recipients, particularly diabetes, have also been associated with shifts in oral microbial composition and an increased susceptibility to periodontal disease (Norrman et al., 2021; Qin et al., 2022). Furthermore, hospitalization-related factors, medication-induced xerostomia, changes in dietary habits, and variations in oral hygiene practices may further influence oral microbial communities and should be considered when interpreting post-transplant microbiological findings (Rajasekaran et al., 2024).

It should also be highlighted that the available studies differ significantly in terms of patient characteristics, immunosuppressive regimens, sample time points, and microbiological techniques, making direct comparisons between them challenging. Furthermore, most published research used relatively small patient groups and documented associations rather than causative correlations, limiting the interpretation of their findings. As a result, the current data should be regarded with caution. More well-designed long-term studies, using standardized microbiological methods, are needed to better identify transplantation-related microbial alterations from those caused by other clinical variables.

Collectively, these observations indicate that alterations in the oral microbiome following organ transplantation are likely multifactorial and reflect the combined effects of transplantation-related interventions and patient-related factors rather than the transplantation procedure alone.

## 6 Conclusions

Organ transplantation has implications beyond improvements in quality of life and may also be associated with alterations in the oral microbiome. Importantly, each of the transplant types discussed in this review is characterized by distinct patterns of oral microbiota changes. In fact, only the changes introduced by kidney and liver transplants appear to be similar in terms of bacteria promoting periodontitis, while the rest of the organs described seem to have a varied impact on the oral microbiome. These findings suggest that, in principle, microbiome analysis may have the potential to differentiate between types of transplanted organs. However, it is worth bearing in mind that there is relatively little research on the impact of lung and liver transplants, and that the dynamics of these microbiological changes in the patient’s oral cavity were highly variable, with intense changes usually occurring within a few days after the transplant. Further studies are needed to better define the oral microbiological changes associated with different types of organ transplantation. Importantly, the observed oral microbiome alterations are likely multifactorial rather than direct consequences of organ transplantation alone. Therefore, potential confounding factors should be carefully considered when interpreting the available evidence and designing future studies.

## References

[B1] Aas J. A. Paster B. J. Stokes L. N. Olsen I. Dewhirst F. E. (2005). Defining the normal bacterial flora of the oral cavity. J. Clin. Microbiol. 43, 5721–5732. doi: 10.1128/JCM.43.11.5721-5732.2005 16272510 PMC1287824

[B2] Abusleme L. Dupuy A. K. Dutzan N. Silva N. Burleson J. A. Strausbaugh L. D. . (2013). The subgingival microbiome in health and periodontitis and its relationship with community biomass and inflammation. ISME J. 7, 1016–1025. doi: 10.1038/ismej.2012.174 23303375 PMC3635234

[B3] Babu R. Kaur M. Sharma S. Sureshan C. S. Gupta R. Baweja S. . (2026). Baseline oral microbiome associated with post-living donor liver transplant early complications within 6 months. Liver Transplant. 32(5):693–707. doi: 10.1097/LVT.0000000000000779 41337711

[B4] Cai Y. Fan Y. Chen A. Wang X. Wang L. Chen J. . (2025). Characteristics of upper and lower respiratory tract microbiota after lung transplantation. Respir. Res. 26, 160. doi: 10.1186/s12931-025-03235-4 40281571 PMC12023598

[B5] Campbell P. M. Willmott T. Humphreys G. J. Piscoran O. Chea H. Summers A. M. . (2023). Transplantation impacts on the oral microbiome of kidney recipients and donors. Front. Microbiomes 2, 1258290. doi: 10.3389/frmbi.2023.1258290 41853371 PMC12993617

[B6] Cekici A. Kantarci A. Hasturk H. Van Dyke T. E. (2014). Inflammatory and immune pathways in the pathogenesis of periodontal disease. Periodontol. 2000 64, 57. doi: 10.1111/PRD.12002 24320956 PMC4500791

[B7] Chenicheri S. R U. Ramachandran R. Thomas V. Wood A. (2017). Insight into oral biofilm: Primary, secondary and residual caries and phyto-challenged solutions. Open Dent. J. 11, 312. doi: 10.2174/1874210601711010312 28839480 PMC5543615

[B8] de Molon R. S. Park C. H. Jin Q. Sugai J. Cirelli J. A. (2018). Characterization of ligature-induced experimental periodontitis. Microsc. Res. Tech. 81, 1412–1421. doi: 10.1002/JEMT.23101 30351474

[B9] Dewhirst F. E. Chen T. Izard J. Paster B. J. Tanner A. C. R. Yu W. H. . (2010). The human oral microbiome. J. Bacteriol. 192, 5002–5017. doi: 10.1128/JB.00542-10 20656903 PMC2944498

[B10] Di Stefano M. Polizzi A. Santonocito S. Romano A. Lombardi T. Isola G. (2022). Impact of oral microbiome in periodontal health and periodontitis: A critical review on prevention and treatment. Int. J. Mol. Sci. 23, 5142. doi: 10.3390/IJMS23095142 35563531 PMC9103139

[B11] Fakhruddin K. S. Ngo H. C. Samaranayake L. P. (2019). Cariogenic microbiome and microbiota of the early primary dentition: A contemporary overview. Oral. Dis. 25, 982–995. doi: 10.1111/odi.12932 29969843

[B12] Farias B. C. Souza P. R. E. Ferreira B. Melo R. S. A. MaChado F. B. Gusmão E. S. . (2012). Occurrence of periodontal pathogens among patients with chronic periodontitis. Braz. J. Microbiol. 43, 909–916. doi: 10.1590/S1517-83822012000300009 24031906 PMC3768898

[B13] Gao X. Zhao S. Wang S. Sun Y. Gao C. (2024). Influence of periodontal status on patients undergoing hematopoietic stem cell transplantation: A retrospective analysis. Heliyon. 10(10), e30998. doi: 10.1016/j.heliyon.2024.e30998 38778978 PMC11108988

[B14] Grenier D. (1995). Characterization of the trypsin-like activity of Bacteroides forsythus. Microbiol. (N. Y). 141, 921–926. doi: 10.1099/13500872-141-4-921

[B15] Haffajee A. D. Socransky S. S. (1994). Microbial etiological agents of destructive periodontal diseases. Periodontol. 2000 5, 78–111. doi: 10.1111/J.1600-0757.1994.TB00020.X 9673164

[B16] Hatzinger M. Stastny M. Grützmacher P. Sohn M. (2016). The history of kidney transplantation. Urologe A. 55, 1353–1359. doi: 10.1007/s00120-016-0205-3 27518791

[B17] He X. Tian Y. Guo L. Lux R. Zusman D. R. Shi W. (2010). Oral-derived bacterial flora defends its domain by recognizing and killing intruders—a molecular analysis using Escherichia coli as a model intestinal bacterium. Microb. Ecol. 60, 655. doi: 10.1007/S00248-010-9708-4 20625713 PMC2954290

[B18] Hong Q. Ren Y. Tang X. Mao B. Zhang Q. Zhao J. . (2025). Impact of live Ligilactobacillus salivarius CCFM1332 and its postbiotics on Porphyromonas gingivalis colonization, alveolar bone resorption and inflammation in a rat model of periodontitis. Microorganisms 13, 1701. doi: 10.3390/MICROORGANISMS13071701 40732209 PMC12300987

[B19] Karched M. Bhardwaj R. G. Qudeimat M. Al-Khabbaz A. Ellepola A. (2022). Proteomic analysis of the periodontal pathogen Prevotella intermedia secretomes in biofilm and planktonic lifestyles. Sci. Rep. 12, 5636. doi: 10.1038/s41598-022-09085-0 35379855 PMC8980031

[B20] Kawamoto D. Borges R. Ribeiro R. A. de Souza R. F. Penas Amado P. P. Saraiva L. . (2021). Oral dysbiosis in severe forms of periodontitis is associated with gut dysbiosis and correlated with salivary inflammatory mediators: A preliminary study. Front. Oral. Health 2, 722495. doi: 10.3389/froh.2021.722495 35048045 PMC8757873

[B21] Könönen E. Gursoy M. Gursoy U. K. (2019). Periodontitis: A multifaceted disease of tooth-supporting tissues. J. Clin. Med. 8, 1135. doi: 10.3390/JCM8081135 31370168 PMC6723779

[B22] Ksiazek M. Mizgalska D. Eick S. Thøgersen I. B. Enghild J. J. Potempa J. (2015). KLIKK proteases of Tannerella forsythia: Putative virulence factors with a unique domain structure. Front. Microbiol. 6, 312. doi: 10.3389/fmicb.2015.00312 25954253 PMC4404884

[B23] Kuramitsu H. K. Wang B. Y. (2006). Virulence properties of cariogenic bacteria. BMC Oral. Health 2006 6:1 6, S11. doi: 10.1186/1472-6831-6-S1-S11 16934112 PMC2147595

[B24] Kure K. Sato H. Suzuki J. Itai A. Aoyama N. Izumi Y. (2019). A novel IkB kinase inhibitor attenuates ligature-induced periodontal disease in mice. J. Periodontal Res. 54, 164–173. doi: 10.1111/JRE.12615 30295325

[B25] Lawrence J. R. Korber D. R. Hoyle B. D. Costerton J. W. Caldwell D. E. (1991). Optical sectioning of microbial biofilms. J. Bacteriol. 173, 6558. doi: 10.1128/JB.173.20.6558-6567.1991 1917879 PMC208993

[B26] Li J. Zhao G. Zhang H. M. Zhu F. F. (2023). Probiotic adjuvant treatment in combination with scaling and root planing in chronic periodontitis: a systematic review and meta-analysis. Benef. Microbes 14, 95–108. doi: 10.3920/BM2022.0056 36856123

[B27] Lima B. P. Hu L. I. Vreeman G. W. Weibel D. B. Lux R. (2018). The oral bacterium Fusobacterium nucleatum binds Staphylococcus aureus and alters expression of the staphylococcal accessory regulator sarA. Microb. Ecol. 78, 336. doi: 10.1007/S00248-018-1291-0 30474730 PMC11796435

[B28] Lundmark A. Hu Y. O. O. Huss M. Johannsen G. Andersson A. F. Yucel-Lindberg T. (2019). Identification of salivary microbiota and its association with host inflammatory mediators in periodontitis. Front. Cell. Infect. Microbiol. 9, 464879. doi: 10.3389/fcimb.2019.00216 31281801 PMC6598052

[B29] Marsh P. D. (2005). Dental plaque: Biological significance of a biofilm and community life-style. J. Clin. Periodontol. 32, 7–15. doi: 10.1111/J.1600-051X.2005.00790.X 16128825

[B30] Marsh P. D. Zaura E. (2017). Dental biofilm: ecological interactions in health and disease. J. Clin. Periodontol. 44, S12–S22. doi: 10.1111/JCPE.12679 28266111

[B31] McGinniss J. E. Whiteside S. A. Deek R. A. Simon-Soro A. Graham-Wooten J. Oyster M. . (2022). The lung allograft microbiome associates with pepsin, inflammation, and primary graft dysfunction. Am. J. Respir. Crit. Care Med. 206, 1508–1521. doi: 10.1164/rccm.202112-2786OC 36103583 PMC9757091

[B32] Megrian D. Taib N. Witwinowski J. Beloin C. Gribaldo S. (2020). One or two membranes? Diderm Firmicutes challenge the Gram-positive/Gram-negative divide. Mol. Microbiol. 113, 659–671. doi: 10.1111/mmi.14469 31975449

[B33] Nadell C. D. Xavier J. B. Foster K. R. (2009). The sociobiology of biofilms. FEMS Microbiol. Rev. 33, 206–224. doi: 10.1111/J.1574-6976.2008.00150.X 19067751

[B34] Nagpal R. Yamashiro Y. Izumi Y. (2015). The two-way association of periodontal infection with systemic disorders: An overview. Mediators Inflamm. 2015, 793898. doi: 10.1155/2015/793898 26339142 PMC4539125

[B35] Natalini J. G. Wong K. K. Nelson N. C. Wu B. G. Rudym D. Lesko M. B. . (2024). Longitudinal lower airway microbial signatures of acute cellular rejection in lung transplantation. Am. J. Respir. Crit. Care Med. 209, 1463–1476. doi: 10.1164/rccm.202309-1551OC 38358857 PMC11208954

[B36] Norrman A. E. Tervahartiala T. Sahlberg E. Sorsa T. Ruokonen H. Grönroos L. . (2021). Salivary biomarkers and oral health in liver transplant recipients, with an emphasis on diabetes. Diagnostics (Basel) 11, 662. doi: 10.3390/diagnostics11040662 33916950 PMC8067605

[B37] Olek K. Kuczaj A. Gruca O. Olek M. Ochman M. Przybyłowski P. . (2022). Condition of the oral cavity in patients after heart transplantation: A preliminary report. Ann. Transplant. 27, e937734. doi: 10.12659/AOT.937734 36560867 PMC9793640

[B38] Plachokova A. S. Andreu-Sánchez S. Noz M. P. Fu J. Riksen N. P. (2021). Oral microbiome in relation to periodontitis severity and systemic inflammation. Int. J. Mol. Sci. 22, 5876. doi: 10.3390/ijms22115876 34070915 PMC8199296

[B39] Ponholzer F. Bogensperger C. Krendl F. J. Krapf C. Dumfarth J. Schneeberger S. . (2024). Beyond the organ: lung microbiome shapes transplant indications and outcomes. Eur. J. Cardiothorac. Surg. 66, ezae338. doi: 10.1093/ejcts/ezae338 39288305 PMC11466426

[B40] Posch G. Sekot G. Friedrich V. Megson Z. A. Koerdt A. Messner P. . (2012). Glycobiology aspects of the periodontal pathogen Tannerella forsythia. Biomolecules 2, 467. doi: 10.3390/BIOM2040467 24970146 PMC4030854

[B41] Qin H. Li G. Xu X. Zhang C. Zhong W. Xu S. . (2022). The role of oral microbiome in periodontitis under diabetes mellitus. J. Oral. Microbiol. 14, 2078031. doi: 10.1080/20002297.2022.2078031 35694215 PMC9176325

[B42] Qu S. Gu Y. Hou X. Wei M. Wang M. Su Y. . (2025). Dual associations of gut and oral microbial networks with kidney transplantation. mSystems 10, e0025225. doi: 10.1128/msystems.00252-25 40631858 PMC12363170

[B43] Rajasekaran J. J. Krishnamurthy H. K. Bosco J. Jayaraman V. Krishna K. Wang T. . (2024). Oral microbiome: A review of its impact on oral and systemic health. Microorganisms 12, 1797. doi: 10.3390/microorganisms12091797 39338471 PMC11434369

[B44] Rohmah D. K. Soekanto S. A. Djais A. A. Fragrantia Theodorea C. Fatma D. Sastradipura S. (2025). The role of Veillonella in the pathogenesis of periodontitis: Systematic review. Cakradonya Dental J. 17, 98–106. doi: 10.24815/cdj.v17i2.43973

[B45] Saito T. Ishihara K. Kato T. Okuda K. (1997). Cloning, expression, and sequencing of a protease gene from bacteroides forsythus ATCC 43037 in Escherichia coli. Infect. Immun. 65, 4888–4891. doi: 10.1128/IAI.65.11.4888-4891.1997 9353083 PMC175704

[B46] Sakakibara J. Nagano K. Murakami Y. Higuchi N. Nakamura H. Shimozato K. . (2007). Loss of adherence ability to human gingival epithelial cells in S-layer protein-deficient mutants of Tannerella forsythensis. Microbiol. (Reading) 153, 866–876. doi: 10.1099/MIC.0.29275-0 17322207

[B47] Sampaio-Maia B. Azevedo M. J. Campos J. Patel M. (2022). Oral cavity and Candida albicans: Colonisation to the development of infection. Pathog. 2022 Vol. 11 Page 335 11, 335. doi: 10.3390/pathogens11030335 35335659 PMC8953496

[B48] Schenkein H. A. Papapanou P. N. Genco R. Sanz M. (2020). Mechanisms underlying the association between periodontitis and atherosclerotic disease. Periodontol. 2000 83, 90–106. doi: 10.1111/PRD.12304 32385879

[B49] Schlich T. (2024). “ Contents,” in The Origins of Organ Transplantation: Surgery and Laboratory Science, 1880-1930 (Rochester, NY: Boydell and Brewer), v–vi. Available online at: https://www.degruyterbrill.com/document/doi/10.1515/9781580467674-toc/html (Accessed June 15, 2026).

[B50] Shlomchik W. D. (2007). Graft-versus-host disease. Nat. Rev. Immunol. 7, 340–352. doi: 10.1038/nri2000 17438575

[B51] Socransky S. S. Haffajee A. D. (2005). Periodontal microbial ecology. Periodontol. 2000 38, 135. doi: 10.1111/J.1600-0757.2005.00107.X 15853940

[B52] Sotozono M. Kuriki N. Asahi Y. Noiri Y. Hayashi M. Motooka D. . (2021). Impact of sleep on the microbiome of oral biofilms. PloS One 16, e0259850. doi: 10.1371/JOURNAL.PONE.0259850 34882696 PMC8659294

[B53] Verma D. Garg P. K. Dubey A. K. (2018). Insights into the human oral microbiome. Arch. Microbiol. 200, 525–540. doi: 10.1007/S00203-018-1505-3 29572583

[B54] Willis J. R. Gabaldón T. (2020). The human oral microbiome in health and disease: From sequences to ecosystems. Microorganisms 8, 308. doi: 10.3390/MICROORGANISMS8020308 32102216 PMC7074908

[B55] Wirth U. Jiang T. Schardey J. Kratz K. Li M. Schirren M. . (2023). The role of microbiota in liver transplantation and liver transplantation-related biliary complications. Int. J. Mol. Sci. 24, 4841. doi: 10.3390/ijms24054841 36902269 PMC10003075

[B56] Xiang X. Peng B. Liu K. Wang T. Ding P. Li H. . (2023). Association between salivary microbiota and renal function in renal transplant patients during the perioperative period. Front. Microbiol. 14, 1122101. doi: 10.3389/fmicb.2023.1122101 37065138 PMC10090686

[B57] Xiang X. Peng B. Liu K. Wang T. Ding P. Zhu Y. . (2024). Prediction of delayed graft function by early salivary microbiota following kidney transplantation. Appl. Microbiol. Biotechnol. 108, 402. doi: 10.1007/s00253-024-13236-w 38951204 PMC11217047

[B58] Yamashita Y. Takeshita T. (2017). The oral microbiome and human health. J. Oral. Sci. 59, 201–206. doi: 10.2334/josnusd.16-0856 28637979

[B59] Yuzefpolskaya M. Bohn B. Ladanyi A. Khoruts A. Colombo P. C. Demmer R. T. (2023). Oral and gut microbiome alterations in heart failure: Epidemiology, pathogenesis and response to advanced heart failure therapies. J. Heart Lung Transplant. 42, 291–300. doi: 10.1016/j.healun.2022.12.009 36586790

[B60] Zaura E. Brandt B. W. de Mattos M. J. T. Buijs M. J. Caspers M. P. M. Rashid M. U. . (2015). Same exposure but two radically different responses to antibiotics: Resilience of the salivary microbiome versus long-term microbial shifts in feces. mBio 6, e01693-15. doi: 10.1128/MBIO.01693-15 26556275 PMC4659469

[B61] Zhang M. Liu Y. Afzali H. Graves D. T. (2024). An update on periodontal inflammation and bone loss. Front. Immunol. 15, 1385436. doi: 10.3389/FIMMU.2024.1385436 38919613 PMC11196616

[B62] Zhou Y. Zhang C. Deng Y. Lei L. Hu T. (2025). Oral microecological community- Streptococcus mutans dysbiosis and interaction provide therapeutic perspectives for dental caries. Arch. Oral. Biol. 178, 106367. doi: 10.1016/j.archoralbio.2025.106367 40812045

[B63] Zorba M. Melidou A. Patsatsi A. Ioannou E. Kolokotronis A. (2020). The possible role of oral microbiome in autoimmunity. Int. J. Womens Dermatol. 6, 357. doi: 10.1016/J.IJWD.2020.07.011 33898698 PMC8060669

